# A Potential Plasmonic Biosensor Based Asymmetric Metal Ring Cavity with Extremely Narrow Linewidth and High Sensitivity

**DOI:** 10.3390/s21030752

**Published:** 2021-01-22

**Authors:** Tianping Xu, Zhaoxin Geng, Yue Su

**Affiliations:** 1 College of Science, Minzu University of China, Beijing 100081, China; 19301346@muc.edu.cn; 2 School of Information Engineering, Minzu University of China, Beijing 100081, China; 3 State Key Laboratory for Integrated Optoelectronics, Institute of Semiconductors, Chinese Academy of Sciences, Beijing 100083, China; suyue@semi.ac.cn

**Keywords:** plasmonic biosensor, asymmetric, narrow linewidth, sensitivity

## Abstract

To achieve high sensitivity and multi-mode sensing characteristics based on the plasmon effect, we explored a high-sensitivity refractive index sensor structure with narrow linewidth and high absorption characteristics based on theoretical analysis. The sensor structure is composed of periodic asymmetric ring cavity array, spacer layer and metal thin-film layer. The reflection spectrum of this structure shows six resonance modes in the wavelength range from visible to near-infrared. The sensor performance was optimized based on the change of the sensor structure parameters combining the simulation data, and the results shown that this kind of asymmetric laminated structure sensor has good sensing performance. In theory, it can be combined with microfluidic technology to achieve sensing detection of diverse test samples, multi-mode and multi-component, which has great potential in the field of biosensing.

## 1. Introduction

Surface plasmons are important and widely used in photolithography [1,2,3,4,5,6], surface-enhanced Raman scattering (SERS) [7,8,9,10], surface-enhanced infrared absorption (SEIRA) [11], photocatalysis [12,13], and other fields due to their excellent optical properties. It propagates at the interface of the metal and medium. It is essentially an electromagnetic oscillation phenomenon caused by the energy coupling of external incident light and free electrons on the metal surface. When electromagnetic oscillation occurs, the nearby electric field will increase significantly, and surface plasmon polarons (SPPs) are extremely sensitive to changes of the surrounding refractive index. Because the changes will affect the propagation constant of SPP and the position of the resonance peak of the evanescent wave. Furthermore, another important application based on surface plasmons is sensing in the fields of biomedicine, chemical detection, food safety, etc. [14,15]. Most surface plasmon sensors are built on the surface plasmon resonance (SPR) theory. The SPR sensor relies on a large and expensive prism or grating coupling system, which is not conducive to the low-cost and integrated development of the sensor. Sensors based on localized surface plasmon resonance (LSPR) seem to be able to adapt to development trends: miniaturization and integration. However, due to the influence of radiation attenuation, the spectrum of the LSPR sensor has the characteristics of wide linewidth and relatively low peak-to-peak, which hinders the improvement of its sensitivity, resulting in limited applications.

It has been proved that when LSPR occurs, the spectral shape of the metal nanostructure is regulated by the geometry of the structure [16,17]. The shape and size of nanostructure can adjust the resonance intensity and line-width, and the relative position between adjacent structures can adjust the interaction of resonance modes. Therefore, the combination parameters of the metal structure can be adjusted artificially to modify the shape, intensity, and position of the resonance wavelength to adapt to specific experiments and applications. For example, in 2019, V.G. Kravets et al. pointed out that plasma surface lattice resonance (SLR) can effectively improve the resonance intensity while reducing the line width [18]. When the light-beam projected on the array, the energy is not absorbed by the unit structure due to scattering and can be absorbed again by the adjacent structure, reducing the radiation loss. Meanwhile, the adjacent structure can interact and produce energy coupling. This causes greater resonance intensity and narrower linewidth than LSPR based on single nanoparticles. In 2017, Liang et al proposed a nested combination structure of a circular hole and a disk, and the disk is located in the center of the circular hole [19]. Due to the strong coupling of the dipole mode between the nanopore and the nano-disk, a plasmonic resonance of the sub-radiation lattice is triggered. This makes its extinction spectrum have a high peak-to-peak ratio. Symmetry breaking is also a very important method to improve the plasmonic resonance [16]. Compared with highly symmetric nanostructures, asymmetric nanostructures make higher-order multipolar modes more active, which is of great significance to improve the sensing performance of the refractive index sensor.

There are two important indicators to measure the performance of the sensor, one is sensitivity and the other is FOM (figure of merit) value. Sensitivity (*S*) is determined by the offset of the resonance wavelength (*Δλ*) and the variation of the refractive index (*Δn*) at the interface, defined as the ratio of the offset of the resonance wavelength to the variation of the ambient refractive index [20]: S=Δλ/Δn. Where the unit of *Δλ* is the nm; the unit of *Δn* is the RIU; the unit of *S* is the nm/RIU (nanometers of peak shift per refractive index unit). The figure of merit (FOM) is determined by the sensitivity and the resonance line width (*d*, the unit: nm), defined as the ratio of sensitivity to resonance line width [20]: FOM=S/d, which is widely used to characterize the sensing capabilities of LSPR biosensor.

To make up for the shortcomings of the traditional LSPR sensor’s wide line width and expand its application range, we have proposed a sensing platform with multi-mode, ultra-high absorption rate, high sensitivity, and high FOM value. The structure of the sensing platform is composed of an asymmetric nanoring cavity array, a quartz spacer layer and a metal thin-film layer. This structure can simultaneously excite propagating surface plasmon (PSP), localized surface plasmon (LSP), and high-order multipole modes, which can produce six resonance modes in the visible to the near infrared region. It provides ideas for designing a high-sensitivity and high-FOM sensing platform. Combined with the simulation data, it is discussed how the sensor structure based on different structural parameters affects the reflectance spectrum and the sensitivity of the sensing platform.

## 2. Structure Design and Theory Analysis

**Figure 1:**
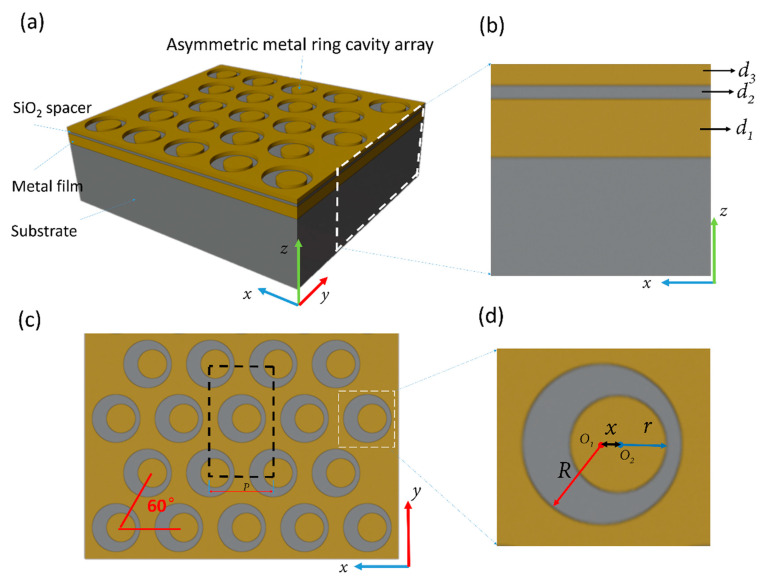
Schematic diagram of the structure we designed. (**a**) 3D view of the structure. The four layers from bottom to top are glass substrate, metal film, SiO_2_ spacer layer, and asymmetric metal ring cavity array. (**b**) Front view of the structure (as shown in the white dotted box in (a)). *d_1_* and *d_2_* are the thickness of the metal film and SiO_2_ spacer. (**c**) Top view of the structure. The black dotted rectangle is the simulation area, and the red line is the period and the angle of period change. (**d**) Some parameters of the structure. *r* is the radius of the disk, *R* is the radius of the hole, and *x* is the center distance between the hole and the disk.

The designed sensing element is a typical sandwich (metal nanostructure/medium/metal thin-film) structure, which is composed of the asymmetric metal ring cavity arrays, SiO_2_ spacer, and metal thin-film from top to bottom (shown in Figure 1). The entire structure is placed on a glass substrate and has the characteristics of periodic changes in the *x*- and *y*-direction. The thickness (*d_3_*) and period (*p*) of the surface asymmetric metal ring cavity is 50 nm and 650 nm, respectively. The direction angle of the period change is 60 degree (as shown in the red line in Figure 1c). The radius (*R*) of the circular hole is 250 nm. The radius (*r*) of the disk is 150 nm. The distance (*x*) between the center of the nanohole (*O*_1_) and the center of the nanodisk (*O_2_*) is 50 nm. The thickness of the metal film (*d_1_*) and SiO_2_ spacer (*d_2_*) is 130 nm and 30 nm, respectively. 

The finite difference time-domain method (FDTD) was used to calculate the reflection spectrum and electric field distribution. (See “Simulation software introduction” in the supplementary materials.) The plane light radiates downward from above the structure along the *z*-direction with a polarization direction parallel to the *x*-direction. Because the structure exhibits periodic characteristics and the angle of periodic change is 60 degrees, we take the black dotted rectangular box as the minimum calculation unit (as shown in Figure 1c) whose side lengths are 1 and $\sqrt{3}$ times of the period, respectively. Both boundaries at the x- and y-directions were set to periodic boundary condition, and the *z*-direction was set to PML boundary condition. The refractive index of the analytes covering the surface of the structure was replaced by the background environment in the calculation. The change of the background refractive index was used to control the change of the refractive index of the analyte. The structure was placed in water environment (background refractive index: 1.33) by default. Here we set the material of the asymmetric metal ring cavity and the metal film to gold.

**Figure 2:**
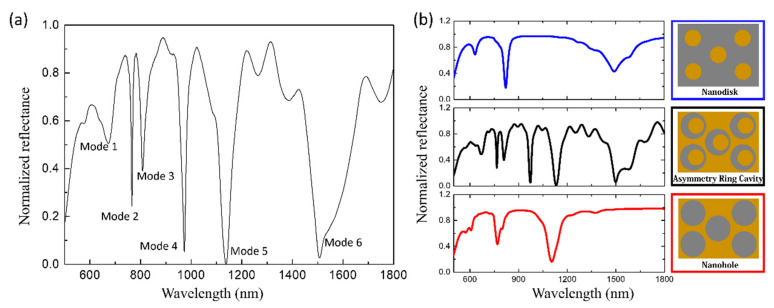
(**a**) The reflection spectrum of the designed structure. (**b**) The reflection spectrum of the nanodisk array (blue line) and the nanohole array (red line) structures which are deviated from the asymmetry ring cavity array. The illustrations are the schematic diagrams of the upper nanostructures corresponding to the spectrums. The environmental refractive index is 1.33.

Figure 2 shows the reflection spectrums of the structure and its components in the wavelength range of 500 nm to 1800 nm. The spectrum in Figure 2a shows six main resonance reflection modes, which located at 668 nm (mode 1), 766 nm (mode 2), 809 nm (mode 3), 972 nm (mode 4), 1131 nm (mode 5), and 1499 nm (mode 6). Seen from the strong resonance intensity of the resonance peak in the reflection spectrum, the designed structure can effectively excite plasmon resonance. The incident energy is coupled to the LSP through a single asymmetric ring cavity, and the latter interacts with the adjacent asymmetric ring cavity through the PSP in the upper space. In addition, the coupling between LSP and PSP enhances the energy of incident light transmitted to SPPs. Meanwhile, due to the asymmetry of the ring cavity, the narrow dark mode and the wide bright mode interact, resulting in the generation of Fano resonance. This makes the resonant peak have a narrow linewidth. The incident light also excites high-order multipole modes, and the overlap and coupling between different modes lead to extremely strong resonance. The results illustrate that the reflectivity of this structure at resonance frequency point is very low.

To explore the reasons for the formation of the spectral shape, we also decomposed the upper nano-asymmetric ring cavity array into nanodisk array and nanohole array and studied them separately. Two array structures were individually combined with the SiO_2_ spacer layer and the gold film to form a new whole. Then two new wholes separately under the same parameter settings and the same conditions were investigated. The corresponding reflection spectra of the two structures composed of nanodisks (blue line) and nanoholes (red line) were shown in Figure 2b. The corresponding electric field distribution of nanodisk and nanohole were shown in supporting information (Figure S1: The electric field diagram of the structure whose upper nanostructure is nanodisk array and Figure S2: The electric field diagram of the structure whose upper nanostructure is nanohole array). Figure 2b confirmed that all resonance modes except mode 4 are the superposition and hybridization of the nanodisk array and nanohole array structures. However, there was a slight deviation between the position of the characteristic peaks of the disassembled structures (nanodisk array and nanohole array) and corresponding peaks of the original structure. This is considered to be that the resonances between the disks and the holes affect each other, causing the resonance peaks to shift from the original. This superposition of resonance modes of the structures with nanodisk array and nanohole array not only retains the characteristics of the two but also successfully suppresses the radiation loss and strengthens the energy coupling after the combined asymmetric ring cavity structure is formed so that the combined spectral characteristic peaks are larger and have a narrower line width. Mode 4 is inspired by the breaking of the symmetry of the cavity formed by the disk and the hole (see Figure S3: The corresponding reflection spectrum when the disks relatively shift from the holes to the right by different distances). Therefore, before combining and forming an asymmetric structure, mode 4 will not appear in the spectrum. The method of dividing the whole into parts used here provides a reference for researchers who are going to design the structure of plasma sensors.

**Figure 3:**
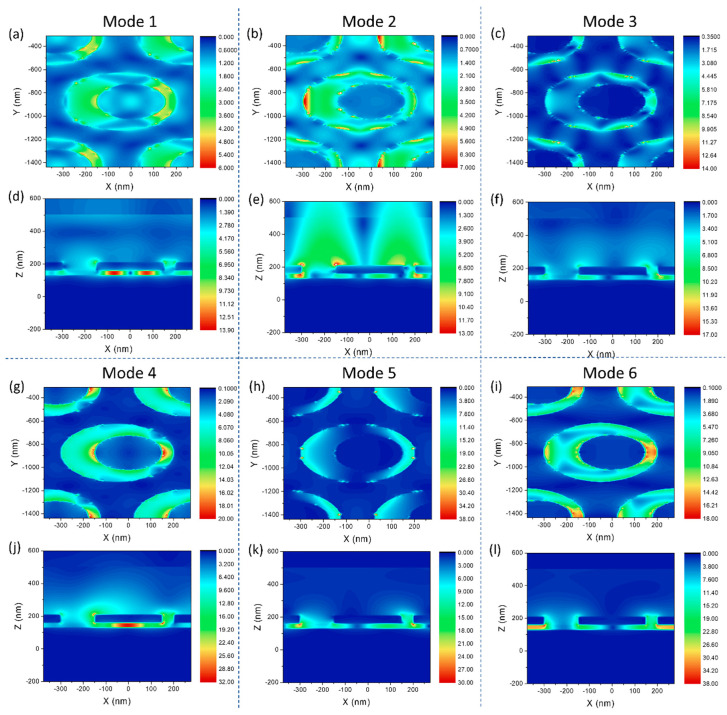
Electric field distribution at the resonance wavelengths of the six modes. (**a**–**c**,**g**–**i**), respectively, are the electric field distributions of six modes (668 nm, 766 nm, 809 nm, 972 nm, 1131 nm, and 1499 nm) on the *x–y* plane of a single asymmetric ring cavity. (**d**–**f**,**j**–**l**), respectively, are the electric field distribution of six modes (668 nm, 766 nm, 809 nm, 972 nm, 1131 nm, and 1499 nm) on the *x–z* plane of a single asymmetric ring cavity.

To better understand the plasmon resonance excited on the structure, the electric field distributions also were calculated at the resonance wavelength in the six modes. Figure 3a–c,g–i, respectively, show the electric field distribution of six modes on the *x–y* plane of a single asymmetric ring cavity. Figure 3d–f,j–l, respectively, show the electric field distribution of six modes on the *x–z* plane of a single asymmetric toroidal cavity.

The asymmetric ring cavity array on the upper surface of the structure can be regarded as an alternative two-dimensional grating. This grating converts the energy of incident light into PSP, making the resonance intensity of mode1 not strong enough. This can be seen from Figure 3d. The electric field distribution tells us the existence of two different resonance components. Figure 3d,f,j show that modes 1, 3, and 4 are all results of the coupling of LSP and PSP, and as the wavelength increases, the ratio of PSP to LSP gradually decreases. When the wavelength is increased to mode 4 and 5, the electric field is bound to the surface of the structure, and the plasmon resonance at this time is mainly composed of LSP. Compared with the other five modes, mode 2 has the least LSP component, and the electric field attenuation distance is large (Figure 3e). For mode 2, the hot spots are mainly concentrated on the surface corners of the asymmetric circular cavity, which is very advantageous for biosensing combined with surface modification.

## 3. Results and Discussion

### 3.1. Influence of Structural Parameter Variation on Sensing Characteristics

The purpose of designing this structure is to obtain a plasmonic-based sensing platform with a simple structure that can provide stable high sensitivity and high FOM value. Therefore, the pros and cons of this structure ultimately need to be understood by applying it to sensing. The sensing principle of this refractive index sensor is very simple: When the refractive index of the analyte near the surface of nano-asymmetric ring cavity structure changes, the position of the spectral resonance peak will shift according to refractive index changing. A sensor with good performance should exhibit a significant large resonance peak shift for refractive index changes. There are two factors to evaluate the performance of this sensors. One is sensitivity and the other is FOM value.

**Figure 4:**
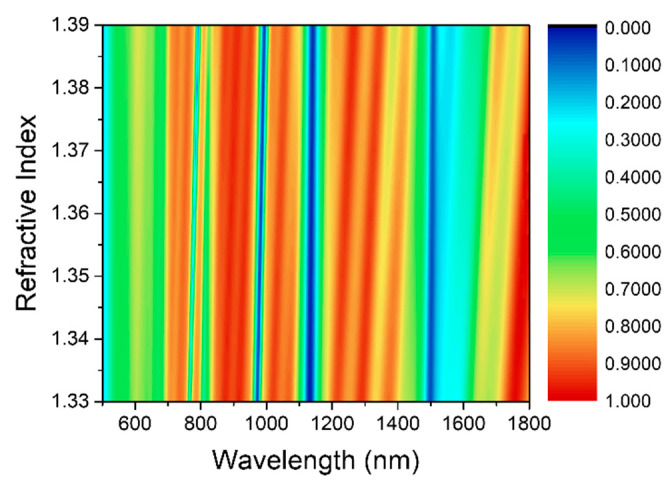
The reflectance spectrum of this structure in the range of analyte refractive index changes from 1.33 to 1.39, where the color represents the numerical value of the reflectivity.

For the case (standard parameters): *p* = 650 nm, *R* = 500 nm, *r* = 300 nm, *x* = 50 nm, *d_1_* = 130 nm, *d_2_* = 30 nm, and *d_3_* = 50 nm, the reflective spectra of designed structures were explored in the wavelength range of 500 to 1800 nm. The contour map (Figure 4) briefly illustrate that the resonance wavelength of modes changes linearly with change of refractive index from 1.33 to 1.39. In Figure 4, the colors change sharply, and red and blue alternately appear, which indicate that each resonance mode has a very large peak-to-valley ratio. Meanwhile, the monochromatic lines are narrow and thin, which also shows from the side that the resonance peaks generated by designed structure have the characteristics of narrow line width, and at λ = 980 nm, 1120 nm, and 1500 nm, the color in the contour map is shown as blue, indicating that designed structure has a very strong resonance intensity at corresponding wavelength and an extremely high absorption rate.

**Figure 5:**
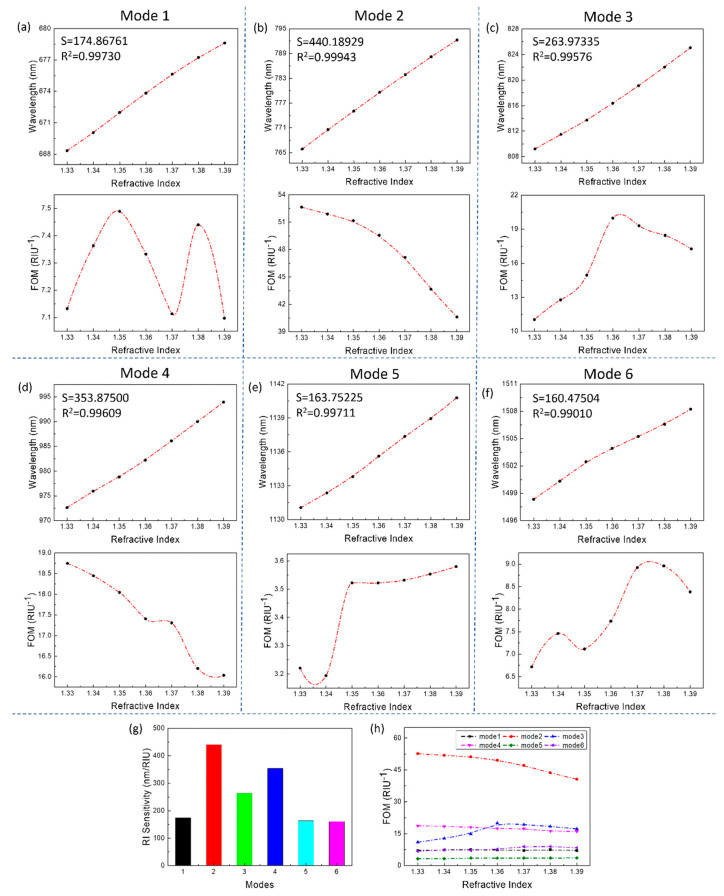
The sensing performance of the designed structure under standard parameters. (**a**–**f**) The graphs of the sensitivity and figure of merit (FOM) relative to the refractive index changes of all modes, respectively. (**g**) Histogram of the sensitivity of each mode. (**h**) Comparison of the FOM of each mode. (The *S* and *R^2^* in the upper left corner are calculated based on linear fitting. The dashed line connecting the data points is simply connected with a smooth curve for readers as a guide for an eye.).

To more intuitively show the sensing performance of each mode, the sensitivity and FOM for the changes of the refractive index were presented in Figure 5. Seen from the Figure 5a–f, R^2^ of sensitivity reach more than 0.99, which show good linearity for the multiple modes. Especially, the mode 2 not only has a sensitivity of 440 nm/RIU but also has a FOM value of up to 52.6, which means that mode 2 is very suitable for biosensing. The resonance wavelengths of all modes have good linear growth with the increase of the refractive index, and the numerical points are basically on a straight line. As for the FOM value of all modes, when the refractive index increases, the FOM value changes without obvious regularity. This is because the increase of the refractive index causes the full width at half maximum (FWHM) of the resonant peaks of some modes to show an increasing trend, making the FOM decrease; others show a decreasing trend, making the FOM increase, and the increase or decrease of the FWHM of the resonant peak mainly depends on its excitation mechanism.

The sensitivity of a refractive index sensor based on (local) surface plasmon resonance is positively correlated with the (local) electric field intensity excited by its nanostructure [21]. Therefore, when the other conditions are the same, the stronger the electric field, the higher the sensor sensitivity. The electric field intensity excited by mode 1 and 3 is small and the part directly related to the refractive index change is too small, which makes the sensing ability of these two modes limited. The electric field intensity excited by mode 1 and 3 is small and the part directly related to the refractive index change is too small, which makes the sensing ability of these two modes limited. Although the electric field intensity excited by modes 5 and 6 is large and localized, it is mainly distributed in the quartz spacer layer and hardly comes into direct contact with the environmental medium. This is not conducive to sensing and detection, which is the fundamental reason why the sensitivity of mode 5 and 6 is not even as good as that of mode 1. The electric field intensity corresponding to mode 2 is not large, and since PSP occupies a larger component in the excitation mechanism, the locality of the electric field is also general. However, most of the electric field distribution of Mode 2 coincides with the environmental medium, which provides it with great potential for refractive index sensing. Mode 4 has the same situation as modes 5 and 6. However, relatively speaking, a part of the electric field excited by mode 4 is distributed at the corners of the asymmetric ring cavity on the surface of the sensor. This part of the electric field coincides with the environmental medium, and the intensity is objective, so that mode 4 also has a good sensitivity. 

From the data point of view (Figure 5), the mode 2 is the most suitable for sensing (sensitivity is 440.2 nm / RIU, FOM = 52.6), followed by the mode 4 (sensitivity is 353.9 nm/RIU, FOM = 18.7). The mode 2 achieves higher sensing performance than that of other plasmonic biosensors [22,23,24,25,26,27,28]. Although mode 4 is slightly inferior to mode 2 in sensitivity and FOM value, it is undeniable that mode 4 is also a resonance mode that is very suitable for sensing. Moreover, the resonance wavelengths of mode 2 and mode 4 are in the middle and low bands, and the requirements for the light source are relatively low, which reduces the cost and facilitates a large-scale promotion. Therefore, in addition to using mode 2 or mode 4 for sensing alone, combining both modes (2 and 4) simultaneously were used for biosensing. The mode 2 and 4 are for comparison and reference with each other, which improves the accuracy and stability of sensing to a certain extent. The modes 3 and 5 are not suitable for signal transmission compared to the mode 2 and 4. Because the sensitivity the modes 3 and 5 is not high enough, and their peak shapes will be affected by heterozygous peaks and change when the refractive index changes. The mode 1 and 6 are limited by their lower sensitivity; therefore, they were difficult to use in biosensing, especial for the sample with a small change in refractive index.

In general, the sensing sensitivity of plasmonic sensing based devices is closely related to the structural parameters. Therefore, to obtain the best sensing performance, several aspects will be optimized. For example, the change of the structure of the sensitive element can cause the shape of the spectrum and the position of resonance peak to change. Here, we take the mode 2 as the optimized direction to investigate the influence of structural parameters on sensing performance. The control variable method was used to optimize the parameters for designed structure to be the best. All the parameters were set for different structures as shown in Table 1.

#### 3.1.1. Influence of the Thickness of Au Film on the Sensitivity and FOM

**Figure 6:**
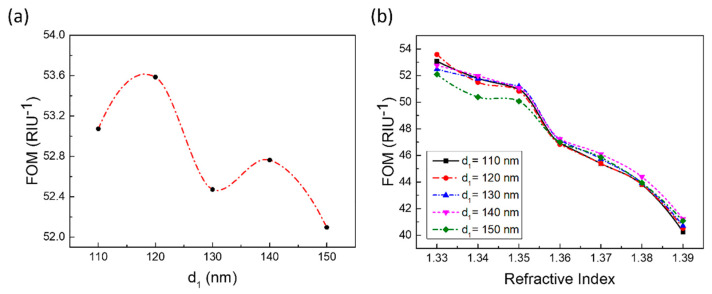
FOM change with the thickness of metal film or environmental refractive index (*n*). (**a**) FOM change with *d*_1_ @ *n* = 1.33. (**b**) FOM change with environmental refractive index @ *n* = 1.33~1.39 and *d_1_* = 110~150 nm. (The solid line and dashed line connecting the data points is simply connected with a smooth curve for readers as a guide for an eye.).

For mode 2, changing the thickness of the metal film (*d_1_*) to explore the sensing properties (sensitivity and FOM) of the designed structure. Under the same environmental refractive index conditions, the position of the resonance peak almost did not shift; however, the intensity and shape would change slightly if only changing *d_1_*, which illustrated that the sensitivity did not increase or decrease with *d_1_* change. Meanwhile, there was a slight change in FWHM. FOM changed with the *d_1_* as shown in Figure 6. Figure 6a shown that FOM maintained at a high and stable level. The maximum FOM difference does not exceed 2. FOM reached maximum value 53.4 when *d_1_* = 120 nm. Seen from Figure 6b, FOM decreased with increasing of environmental refractive index (*n*). The variation trend of FOM of different *d_1_* is consistent.

#### 3.1.2. Influence of the Thickness of the SiO_2_ Spacer Layer on the Sensitivity and FOM

**Figure 7:**
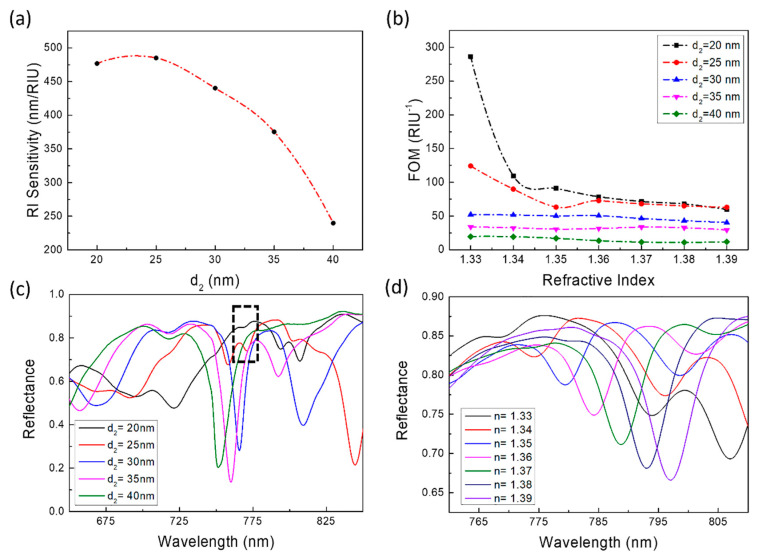
The performance influenced by *d*_2_. (**a**) The sensitivity changes with *d*_2_. (**b**) The impact of *d*_2_ changes on FOM, when *n* = 1.33–1.39. (The dashed line connecting the data points is simply connected with a smooth curve for readers as a guide for an eye.) (**c**) When *n* = 1.33, the reflection spectrum of the structure changes with *d*_2_. Mode 2 is in the range of 750–775 nm. (**d**) When *d*_2_ = 20 nm, the reflection spectrum of the structure changes with refractive index (RI).

The spacer layer (*d*_2_) also played a key role on the sensing performances of designed structure. As shown in Figure 7a, as *d*_2_ gradually increases, the sensitivity shows a downward trend: starting from *d*_2_ = 25 nm, the sensitivity decreased quickly with *d*_2_ increasing. Meanwhile, FOM decreased with *d*_2_ increasing (Figure 7b). The increasing thickness of the SiO_2_ spacer layer would make the distance between the upper asymmetric ring cavity array and the lower Au film increase, which causes the coupling between the upper asymmetric ring cavity array and the lower Au film increase weaken, and the near-field enhancement also weakens. Except for the case of *d*_2_ = 20 and 25 nm, *n* = 1.33, and 1.34, FOM is not sensitive to refractive index changes. The reason why the FOM value is larger when *d*_2_ = 20 and 25 nm, *n* = 1.33 and 1.34 is mode 2 is degraded and the resonance intensity is greatly reduced. However, thanks to this too, the FWHM of the mode 2 is also greatly reduced (as shown in the black dotted box in Figure 7c). However, as the refractive index continues to increase, in the wavelength range of 700 to 800 nm, the resonance peak intensity of mode 2 gradually increases, and the FWHM also increases (Figure 7d). This caused the FOM value to begin to decline and there were two processes of rapid decline and slow decline. Unlike *d*_1_, *d*_2_ has a huge impact on the position, strength, and shape of mode 2.

#### 3.1.3. Influence of the Size of the Asymmetrical Ring Cavity on the Sensitivity and FOM

**Figure 8:**
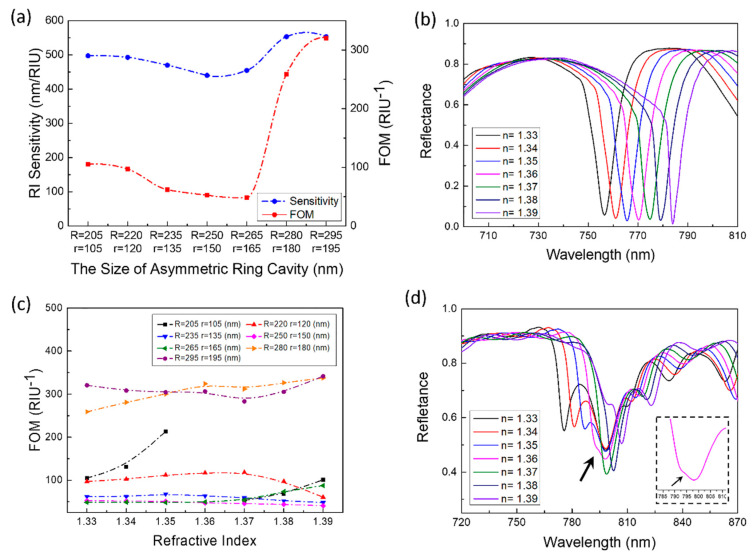
(**a**) The sensitivity and FOM (n = 1.33) changes with *R* and *r*. (**b**) When *R* = 265 nm and *r* = 165 nm, the reflectance spectrum of the sensor with the changes of the refractive index from 1.33 to 1.39. (**c**) The impact of *R* and *r* changes on FOM, when *n* = 1.33–1.39. (The dashed line connecting the data points is simply connected with a smooth curve for readers as a guide for an eye.) (**d**) When *R* = 205 nm and *r* = 105 nm, the reflectance spectrum of the sensor with the changes of the refractive index from 1.33 to 1.39. The inset is a partial spectrum when *n* = 1.36 and mode 2 where the arrow points are very weak, just bringing a slight fluctuation to the spectrum.

The sensing performances of the designed structure were explored through changing the size of the asymmetric ring cavity. The radius of the circular hole and the disc increased at the same time and the increasing value of the two radii were in the same scale. Seen from the results of the Case C (Figure 8), the variable size of the asymmetric ring cavity had a greater impact on the sensing performances (sensitivity and FOM). The sensitivity and FOM both decreased first and then increased with the increasing of *R* and *r* (Figure 8a). The inflection points of the change both *R* and *r* were where the ring cavity size was *R* = 265 nm and *r* = 165 nm. In the process of decreasing the sensitivity, the amplitude was greater than that of FOM; however, in the process of increasing, sensitivity and FOM have large increasing (sensitivity reached about 200 nm/RIU, FOM reached about 300 RIU^−1^). When the size of the ring cavity changed to *R* = 265 nm and *r* = 165 nm, mode 2 shown an "inward depression" shape in the spectrum (Figure 8b). The middle and lower parts of the "inward depression" resonance peak was narrower, which make the shift of the peak less obvious. Figure 8c shown the relationship between the refractive index and the FOM change of the ring cavity with different sizes. When the sizes were *R* = 205 nm, *r* = 105 nm, *n* = 1.36, the resonance peak was extremely small and the half-maximum width was close to 0. Meanwhile, the FOM was very large. Except for the size of *R* = 205 nm and *r* = 105 nm, the FOM of other sizes was not sensitive to the change of the environmental refractive index and the overall FOM was steadily maintained at a large position.

#### 3.1.4. Influence of the Position of the Disks in the Asymmetrical Ring Cavity on the Sensitivity and FOM

**Figure 9:**
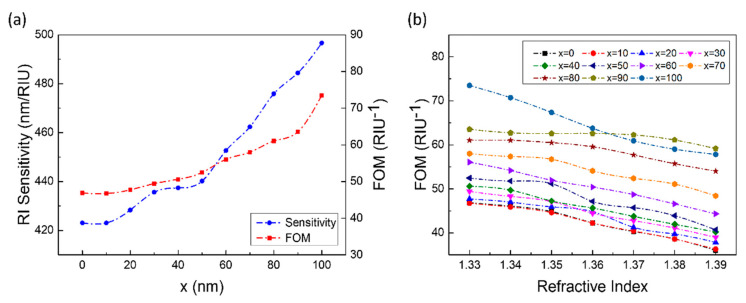
The influence of the distance between the center of a circular hole and a circular disk on the sensing performances. (**a**) The sensitivity and FOM (*n* = 1.33) changes with *x*. (**b**) The impact of *x* changes on FOM when *n* = 1.33–1.39. (The dashed line connecting the data points is simply connected with a smooth curve for readers as a guide for an eye.).

Because the relative position of the disk and the hole was directly related to the degree of asymmetry of the structure, which would affect the sensing performance of the structure. The process of changing the position of the disk starts from the coincidence of the center of the disk and the hole, and finally, the disk and the hole were inscribed. The disk moved 10 nm to the right relative to the position of the hole each time. According to the results of Case D (Figure 9), as the disk continues to move to the right (one side), the symmetry of the structure was gradually destroyed and the sensing performance of the structure was getting better. Both sensitivity and FOM shown an upward trend, and the increase in sensitivity was relatively larger than that of FOM (Figure 9a). On the other hand, during the change of the environmental refractive index from 1.33 to 1.39, although the FOM shown a downward trend overall, the decline was not obvious (Figure 9b). The FOM shows good stability, indicating that the structure could still maintain excellent sensing performance in a complex detection environment. 

The results illustrated that in the sensor structure design stage, appropriately introducing asymmetry to the structure was very helpful to improve the shape of the characteristic peaks in the spectrum and improve the sensing performances. For example, if the ordered array structure was replaced with a disordered array structure, the sensing performance of the sensor may not increase but decrease. Therefore, it was necessary to combine both symmetry and asymmetry to improve the sensitivity.

### 3.2. Influence of the Material of the Structure on the Sensitivity and FOM

**Figure 10:**
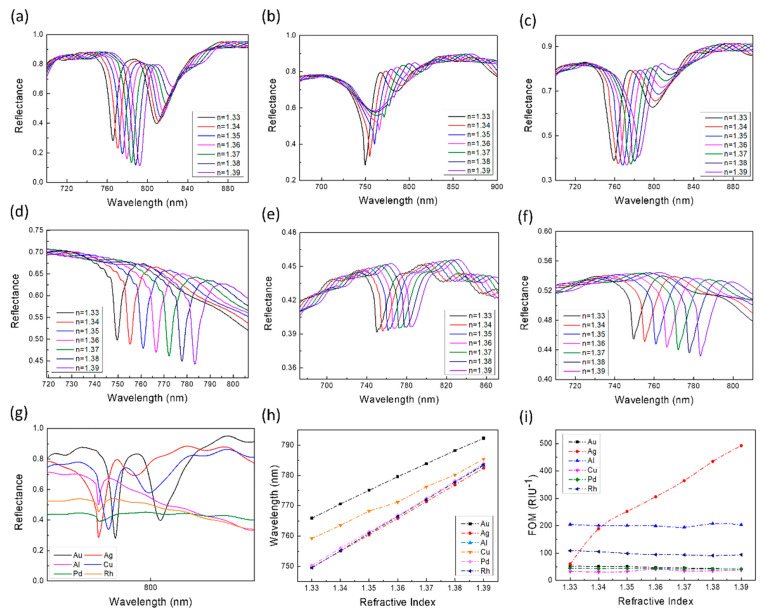
Mode 2 obtained by using different materials under standard structural parameters and within a certain range of refractive index changes: (**a**) Au, (**b**) Ag, (**c**) Cu, (**d**) Al, (**e**) Pd, (**f**) Rh. (**g**) When *n* = 1.33, the spectra of sensors of different materials in the mode 2 band. (**h**,**i**) is the sensitivity and FOM of the sensor by using different materials. (The dashed line connecting the data points is simply connected with a smooth curve for readers as a guide for an eye.).

The most widely used and fully studied material in plasmonic photonics is Au. In addition to Au, Ag, and Cu are also very common materials. These noble metals were initially preferred because of their stability and high electrical conductivity, which caused violent resonances [29]. Al is not a precious metal. Therefore, the cost of Al is relatively low. Al has a relatively high plasma frequency, and because of its abundance, sustainability, and simple processing requirements, Al is considered to be a significant plasma material [30,31]. Rh has also attracted researchers’ attention because of its similar optical properties to Al and its oxidation resistance. There are also some uncommon metals, such as Pd, which have shown good sensitivity to specific gases and have attracted many researchers to explore their properties [29]. Here we explored the influence of different materials on the sensing performance of our sensors. The method of exploration is to fix the standard structural parameters and replace all the parts of the structure that are made of Au with other materials (Ag, Cu, Al, Pd, and Rh). Then, within a certain range of refractive index change (*n* = 1.33–1.39), we calculated the sensing performance of sensors based on different materials. The all results were shown in Figure 10 and Table 2.

Figure 10g illustrated that the material was changed from Au to other metals, mode 2 had a blue shift, and from the waveform point of view, the spectra near mode 2 corresponding to Au, Ag, and Cu are relatively close; while the waveforms of Al, Pd, and Rh are relatively close to each other. In contrast, the peak-to-valley ratios of the top three precious metals are larger and the peak shapes are clearer. The spectra corresponding to the latter three (mode 4, 5, 6) can degenerate from the high-order resonance modes of the former. However, the six materials have a good linear fit for the resonant wavelength, and the refractive index shift (Figure 10h) and their R^2^ are very close to one. Regarding the sensitivity of sensors based on six materials, from large to small, they are Rh, Al, Pd, Ag, Au, and Cu. Except for Cu, the sensitivity of the other four materials are 100 nm/RIU higher than that of Au. On the other hand, there are two types of FOM the changes with the refractive index. The FOM values of the sensors corresponding to Au, Al, Cu, Pd, and Rh are not sensitive to changes in the refractive index of the environment and remain stable overall. The FOM corresponding to Al is as high as about 200 RIU^−1^. The smallest is the FOM corresponding to Cu, which can also reach a size above 30 RIU^−1^. The FOM of the sensor corresponding to Ag changes significantly with the change of the environmental refractive index: when the refractive index is 1.33, the FOM is 59.7 RIU^−1^; when the refractive index is 1.39, the FOM changes to 492.5 RIU^−1^. The magnitude of change is as large as 432.8 RIU^−1^. From the data point of view, in addition to Cu, the replacement of Au with four other materials seems to significantly help improve the sensing performance of the sensor. However, we can find through observation that the peak shape of mode 2 corresponding to Ag changes significantly with the change of refractive index and the peak becomes less and less obvious (Figure 10b). This is because more hybrid peaks appear under a high refractive index [31], and the presence of hybrid peaks will affect mode 2. This is not conducive to the detection and resolution of the instrument, thereby affecting the accuracy of sensing. Although the peak shape corresponding to Rh can maintain the stability of the shape when the refractive index changes, there is a slight fluctuation at the top of the resonance peak (Figure 10e). The size of the fluctuation will also change slightly with the change in the refractive index. This makes it possible for the instrument to detect one or two peaks in one refractive index during detection. This is also detrimental to the accuracy of sensing.

### 3.3. Absorption of the Structure

Generally speaking, the reflectivity of metal will be very high under normal incident light, that is, the absorption rate is very low [32]. In the structure design, we found an interesting phenomenon: the mode 4, mode 5, and mode 6 all have high absorption rates. As shown in Figure 2 and Figure 3, in the reflection spectrum of the designed structure, the three resonance modes located at λ = 975 nm, λ = 1140 nm, and λ = 1499 nm have very strong resonance intensity. The maximal absorption rate of these three absorption peaks can reach 98.88%, 99.96%, and 99.17%, respectively. These are far superior to other non-metamaterial plasma sensors [23,33,34,35,36].

The absorption rate is related to many factors, the most important of which are the internal loss rate (δ) and external leakage rate (γ_e_) of the structure [37]. The external leakage rate is related to the Specific structure. The ultra-high absorption rate of the three absorption peaks is attributed to the tight arrangement of the holes and disk structure and the array structure, which reduces radiation loss and inhibits external leakage. In addition, the incident light successfully excites a strong plasmon resonance, which further improves the absorption of the structure [32]. From another perspective, as a metal-insulator-metal structure, the upper and lower layers of metal will form a magnetic dipole at the resonance wavelength. The magnetic dipole couples with the magnetic component in the incident light to produce magnetic resonance [38]. This is the principle of perfect absorption. This also gave us some inspiration: the structure we designed seems to have the potential to be a perfect absorber, and it can be more widely used in various fields. For example, the perfect absorber can also be used as a refractive index sensor for measurement sensing [38], and this kind of sensor also has the advantages of a simple system (only single-wavelength light source is needed) and is almost not affected by incident angle and polarization state (further detailed analysis is not here). Our design ideas can also provide references for other perfect absorber designs.

## 4. Conclusions

In summary, a high-sensing performance sensor with a narrow linewidth and high absorptivity characteristics has been demonstrated. According to the results, the resonance wavelength shift corresponding to the designed structure has a good linear relationship with the change of the environmental refractive index. By changing the thickness of the Au film (*d_1_*), the thickness of the spacer layer (*d_2_*), the size of the asymmetric ring cavity (*R* and *r*), and the position of the disc of the structure (*x*), we found that our sensor has excellent and stable sensing performance (sensitivity is 553 nm/RIU, FOM is 341.5 RIU^−1^, and the absorption rate is 99.96%). In specific production practices, the structural parameters of the sensor can be adjusted according to specific conditions to better adapt to actual conditions. The structure we designed provides a new idea and a good reference for the design of plasma sensors for biosensing in the future.

## Figures and Tables

**Table 1:** Structural parameters for different cases.

(Unit: nm)	Au Film Thickness (*d*_1_)	SiO_2_ Spacer Layer Thickness (*d*_2_)	Radius of Disks (*r*)	Radius of Round Holes (*R*)	Center Distance of the Circular Hole of The Disc (*x*)
Standard	130	30	150	250	50
Case A	110:10:150 ^1^	30	150	250	50
Case B	130	20:5:40	150	250	50
Case C	130	30	105:15:195	205:15:295	50
Case D	130	30	150	250	0:10:100

^1^ For K_1_: k: K_2_ mentioned in Table 1, K_1_ is the lower limit of value change, K_2_ is the upper limit of value change and k is the magnitude of each value change.

**Table 2:** Sensing performance and accuracy of different material of designed structure.

Metal Materials	Sensitivity	R^2^ of Sensitivity	FOM (Refractive Index = 1.33)	FWHM
Au	440.18929	0.99943	52.64698	8.36115
Ag	546.20252	0.99993	59.71468	89.14687
Cu	429.11429	0.99679	33.28716	12.89128
Al	563.21429	0.99997	205.00363	2.74734
Pd	556.84218	0.99980	45.01732	12.36951
Rh	566.50357	0.99997	108.86442	5.20375

## Data Availability

The data presented in this study are available in the main text and the supplementary material for this article.

## Supplementary Materials

The following are available online at https://www.mdpi.com/1424-8220/21/3/752/s1, Figure S1: The electric field diagram of the structure whose upper nanostructure is nanodisk array, Figure S2: The electric field diagram of the structure whose upper nanostructure is nanohole array, Figure S3: The corresponding reflection spectrum when the disks relatively shift from the holes to the right by different distances, Simulation software introduction.
