# Supplementary material for: A Potential Plasmonic Biosensor Based Asymmetric Metal Ring Cavity with Extremely Narrow Linewidth and High Sensitivity
Source: Sensors (Basel). 2021 Jan 22;21(3):752. doi: 10.3390/s21030752 (PMC7865613; doi:10.3390/s21030752)
Supplement: Supplementary file 1 [file sensors-21-00752-s001.pdf]

A potential plasmonic biosensor based asymmetric metal ring cavity with extremely narrow linewidth and high sensitivity

Tianping Xu [1], Zhaoxin Geng [2, 3*] and Yue Su [3]

[1] College of Science, Minzu University of China, Beijing, 100081, P. R. China; 602063033@qq.com
[2] School of Information Engineering, Minzu University of China, Beijing, 100081, P. R. China; zxgeng@muc.edu.cn
[3] State Key Laboratory for Integrated Optoelectronics, Institute of Semiconductors, Chinese Academy of Sciences, Beijing, 100083, P. R. China; suyue@semi.ac.cn
* Correspondence: zxgeng@muc.edu.cn; Tel.: +86-10-8230-4531

Supporting Information

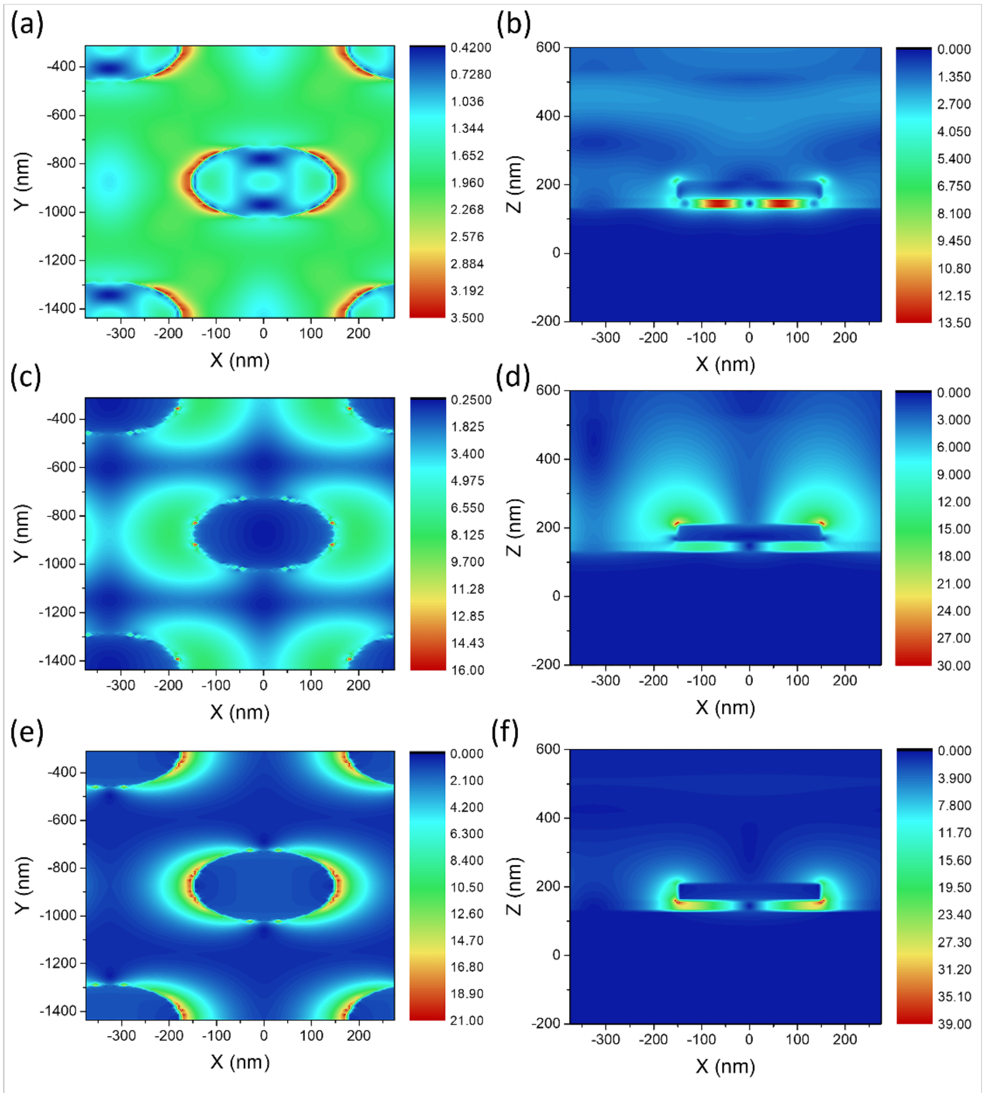

Figure S1. The electric field diagram of the structure whose upper nanostructure is nanodisk array. (a), (c) and (e) respectively are the electric field distributions (631 nm, 821nm and 1491nm) on the *x*-*y* plane in the middle of a single disk. (b), (d) and (f) respectively are the electric field distributions (631nm, 821nm and 1491nm) on the *x*-*z* plane in the middle of a single disk.

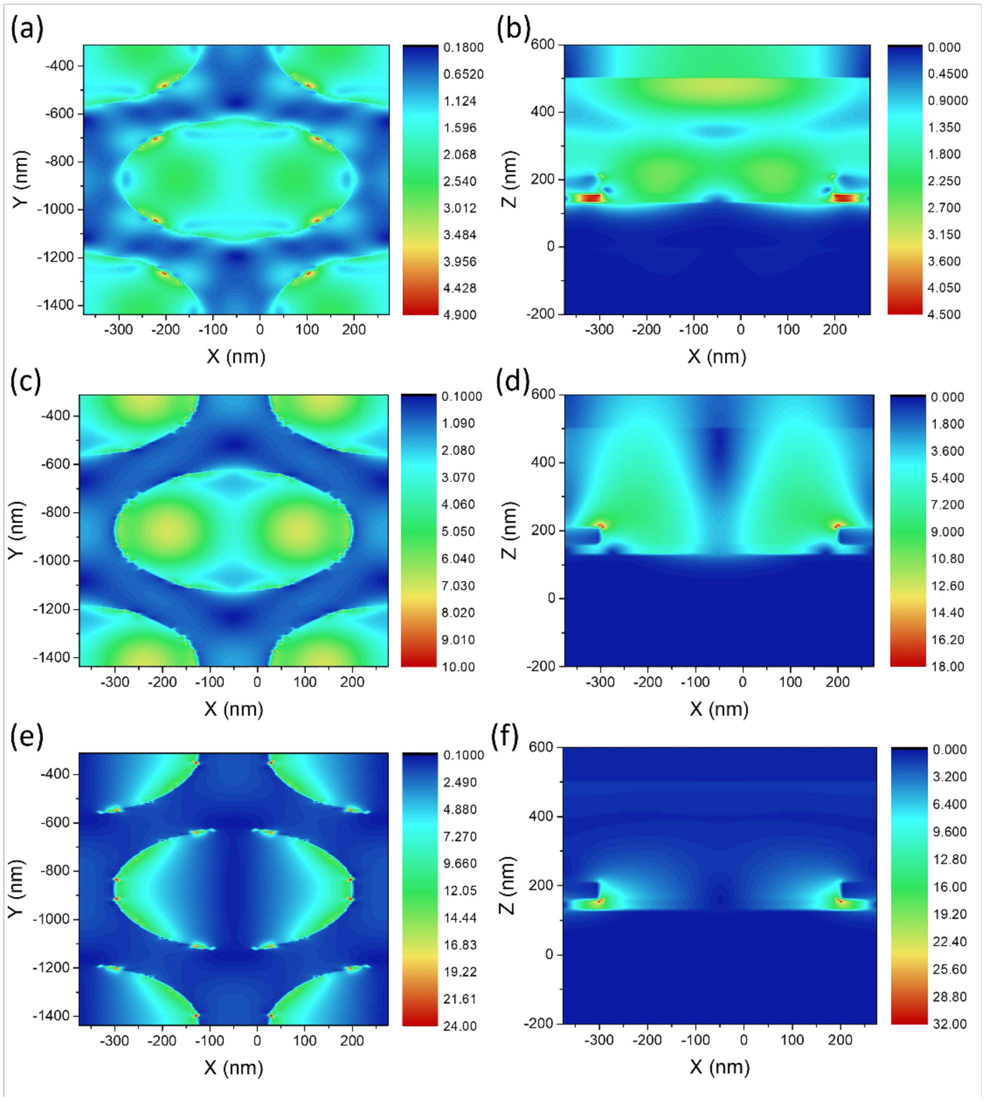

Figure S2. The electric field diagram of the structure whose upper nanostructure is nanohole array. (a), (c) and (e) respectively are the electric field distributions (606nm, 769nm and 1104nm) on the *x*-*y* plane in the middle of a single disk. (b), (d) and (f) respectively are the electric field distributions (606nm, 769nm and 1104nm) on the *x*-*z* plane in the middle of a single hole.

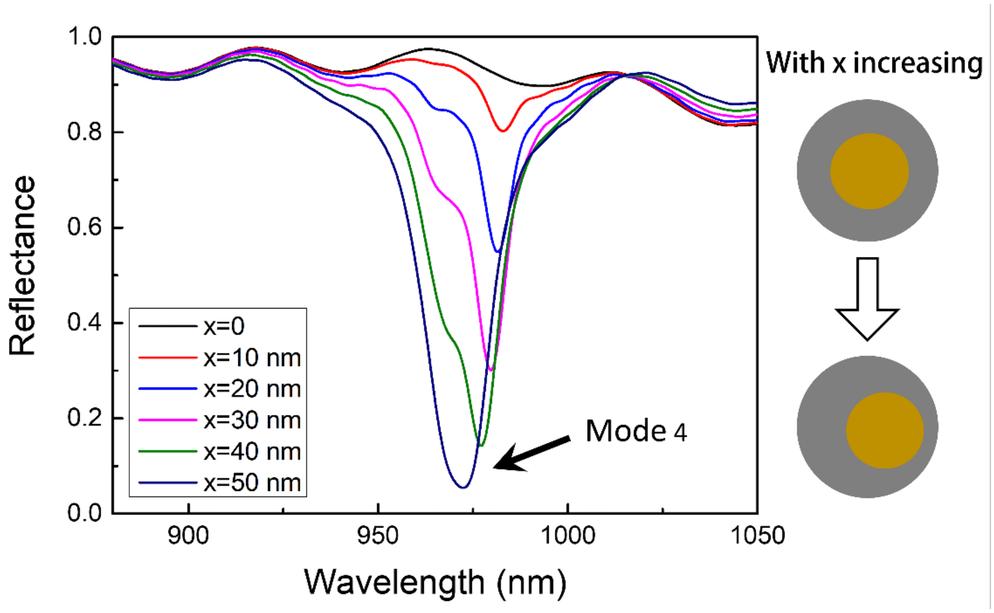

Figure S3. The corresponding reflection spectrum when the disks relatively shift from the holes to the right by different distances

When the nanodisks and nanoholes are combined with the center of the circle coincident (x=0), mode 4 will not be excited because of the completely symmetrical structure. However, as the shifts of the disks along the right direction parallel to the polarization direction of the incident light gradually increase, mode 4 grows from nothing and its peak intensity becomes larger. (When x=50 nm, this combination structure of disk and hole becomes the asymmetric circular cavity mentioned in our article.)

Simulation software introduction

The simulation software we used in this work is FDTD (2019b) from LUMERICAL. FDTD is a simulator within Lumerical's DEVICE Multiphysics Simulation Suite. The simulation principle of the software is to solve Maxwell's equations. By building a structural model and setting the light source, simulation range, boundary conditions and monitors to constrain the simulation conditions, and then combined with a large number of calculations on the computer, we have obtained the desired simulation results.
